# The health impacts of globalisation: a conceptual framework

**DOI:** 10.1186/1744-8603-1-14

**Published:** 2005-08-03

**Authors:** Maud MTE Huynen, Pim Martens, Henk BM Hilderink

**Affiliations:** 1 International Centre for Integrative Studies (ICIS), Maastricht University, Maasticht, The Netherlands; 2 Faculty of Natural Sciences, Open University, Heerlen, The Netherlands; 3 Zuyd University, Heerlen, The Netherlands; 4 Netherlands Environmental Assessment Agency (MNP), Bilthoven, the Netherlands

## Abstract

This paper describes a conceptual framework for the health implications of globalisation. The framework is developed by first identifying the main determinants of population health and the main features of the globalisation process. The resulting conceptual model explicitly visualises that globalisation affects the institutional, economic, social-cultural and ecological determinants of population health, and that the globalisation process mainly operates at the contextual level, while influencing health through its more distal and proximal determinants. The developed framework provides valuable insights in how to organise the complexity involved in studying the health effects resulting from globalisation. It could, therefore, give a meaningful contribution to further empirical research by serving as a 'think-model' and provides a basis for the development of future scenarios on health.

## Introduction

Good health for all populations has become an accepted international goal and we can state that there have been broad gains in life expectancy over the past century. But health inequalities between rich and poor persist, while the prospects for future health depend increasingly on the relative new processes of globalisation. In the past globalisation has often been seen as a more or less economic process. Nowadays it is increasingly perceived as a more comprehensive phenomenon, which is shaped by a multitude of factors and events that are reshaping our society rapidly. This paper describes a conceptual framework for the effects of globalisation on population health. The framework has two functions: serving as 'think-model', and providing a basis for the development of future scenarios on health.

Two recent and comprehensive frameworks concerning globalisation and health are the ones developed by Woodward et al. [1], and by Labonte and Togerson [2]. The effects that are identified by Woodward et al. [1] as most critical for health are mainly mediated by economic factors. Labonte and Torgerson [2] primarily focus on the effects of economic globalisation and international governance. In our view, however, the pathways from globalisation to health are more complex. Therefore, a conceptual framework for the health effects of the globalisation process requires a more holistic approach and should be rooted in a broad conception of both population health and globalisation. The presented framework is developed in the following three steps: 1) defining the concept of population health and identifying its main determinants, 2) defining the concept of globalisation and identifying its main features and 3) constructing the conceptual model for globalisation and population health.

## Population health

As the world around us is becoming progressively interconnected and complex, human health is increasingly perceived as the integrated outcome of its ecological, social-cultural, economic and institutional determinants. Therefore, it can be seen as an important high-level integrating index that reflects the state-and, in the long term, the sustainability-of our natural and socio-economic environments [3]. This paper primarily focuses on the physical aspects of population health like mortality and physical morbidity.

Our identification of the most important factors influencing health is primarily based on a comprehensive analysis of a diverse selection of existing health models (see Huynen et al [4] for more details). We argue that the nature of the determinants and their level of causality can be combined into a basic framework that conceptualises the complex multi-causality of population health. In order to differentiate between health determinants of different nature, we will make the traditional distinction between social-cultural, economic, environmental and institutional factors. These factors operate at different hierarchical levels of causality, because they have different positions in the causal chain. The chain of events leading to a certain health outcome includes both proximal and distal causes; proximal factors act directly to cause disease or health gains, and distal determinants are further back in the causal chain and act via (a number of) intermediary causes [5]. In addition, we also distinguish contextual determinants. These can be seen as the macro-level conditions shaping the distal and proximal health determinants; they form the context in which the distal and proximal factors operate and develop.

**Figure 1:**
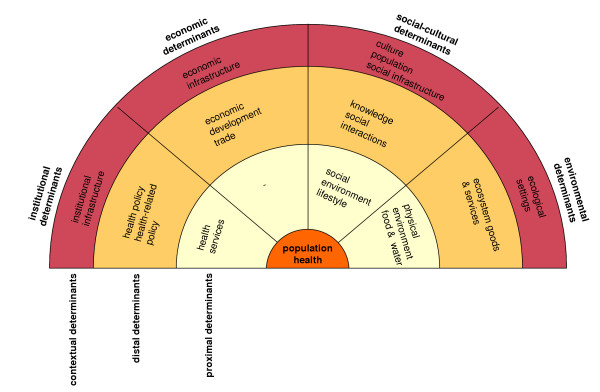
Multi-nature and multi-level framework for population health.

Subsequently, a further analysis of the selected health models and an intensive literature study resulted in a wide-ranging overview of the health determinants that can be fitted within this framework (Figure 1 and Table 1). We must keep in mind, however, that determinants within and between different domains and levels interact along complex and dynamic pathways to 'produce' health at the population level. Additionally, health in itself can also influence its multi-level, multi-nature determinants; for example, ill health can have a negative impact on economic development.

**Table 1:** Determinants of population health

**Level/ Nature**	**General determinants**	**More detailed determinants**
**Contextual level **		
Institutional	Institutional infrastructure	Governance structure Political environment System of law Regulation
Economic	Economic infrastructure	Occupational structure Tax system Markets
Social-cultural	Culture	Religion Ideology Customs
	Population	Population size Structure Geographical distribution
	Social infrastructure	Social organisation Knowledge development Social security Insurance system Mobility and communication
Environmental	Ecological settings	Ecosystems Climate
**Distal level**		
Institutional	Health policy	Effective public health policy Sufficient public health budget
	Health-related policies	Effective food policy Effective water policy Effective social policy Effective environmental policy
Economic	Economic development	Income/wealth Economic equity
	Trade	Trade in goods and services Marketing
Social-cultural	Knowledge	Education and literacy Health education Technology
	Social interactions	Social equity Conflicts Travel and migration
Environmental	Ecosystem goods and services	Habitat Information Production Regulation
**Proximal level**		
Institutional	Health services	Provision of and access to health services
Economic	-	-
Social-cultural	Lifestyle	Healthy food consumption patterns Alcohol and tobacco use Drug abuse Unsafe sexual behaviour Physical activity Stress coping Child care Lifestyle related endogen factors (blood pressure, obesity, cholesterol levels)
	Social environment	Social support and informal care Intended injuries and abuse/violence
Environmental	Food and water	Sufficient quality Sufficient quantity Sanitation
	Physical environment	Quality of the living environment (biotic, physical and chemical factors) Unintended injuries

## Globalisation

There is more and more agreement on the fact that globalisation is an extremely complex phenomenon; it is the interactive co-evolution of multiple technological, cultural, economic, institutional, social and environmental trends at all conceivable spatiotemporal scales. Hence, Rennen and Martens [6] define contemporary globalisation as an intensification of cross-national cultural, economic, political, social and technological interactions that lead to the establishment of transnational structures and the global integration of cultural, economic, environmental, political and social processes on global, supranational, national, regional and local levels. Although somewhat complex, this definition is in line with the view on globalisation in terms of deterritorialisation and explicitly acknowledges the multiple dimensions involved.

However, the identification of all possible health effects of the globalisation process goes far beyond the current capacity of our mental ability to capture the dynamics of our global system; due to our ignorance and interdeterminacy of the global system that may be out of reach forever [7]. In order to focus our conceptual framework, we distinguish-with the broader definition of globalisation in mind-the following important features of the globalisation process: (the need for) new global governance structures, global markets, global communication and diffusion of information, global mobility, cross-cultural interaction, and global environmental changes (Table 2) (see Huynen et al. [4] for more details).

**Table 2:** Features of globalisation

New global governance structure	Globalisation influences the interdependence among nations as well as the nation state's sovereignty leading to (a need for) new global governance structures.
Global markets	Globalisation is characterised by worldwide changes in economic infrastructures and the emergence of global markets and a global trading system.
Global communication and diffusion of information	Globalisation makes the sharing of information and the exchange of experiences around common problems possible.
Global mobility	Global mobility is characterised by a major increase in the extensity, intensity and velocity of movement and by a wide variety in 'types' of mobility.
Cross-cultural interaction	Globalising cultural flows result in interactions between global and local cultural elements.
Global environmental changes	Global environmental threats to ecosystems include global climate change, loss of biodiversity, global ozone depletion and the global decline in natural areas.

## Conceptual model for globalisation and health

**Figure 2:**
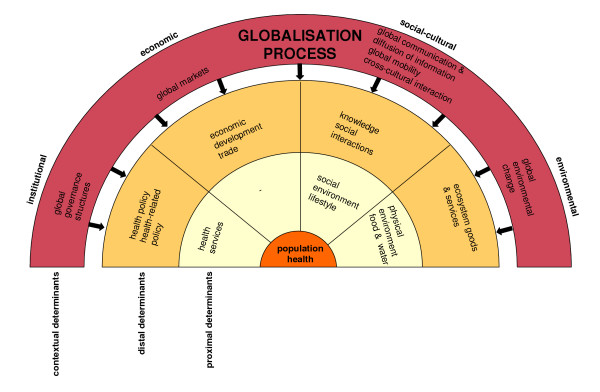
Conceptual framework for globalisation and population health.

We have identified (the need for) global governance structures, global markets, global communication and the diffusion of information, global mobility, cross-cultural interaction, and global environmental changes as important features of globalisation. Based on Figure 1 and Table 1, it can be concluded that these features all operate at the contextual level of health determination and influence distal factors such as health(-related) policies, economic development, trade, social interactions, knowledge, and the provision of ecosystem goods and services. In turn, these changes in distal factors have the potential to affect the proximal health determinants and, consequently, health. Our conceptual framework for globalisation and health links the above-mentioned features of the globalisation process with the identified health determinants. This exercise results in Figure 2.

**Figure 3:**
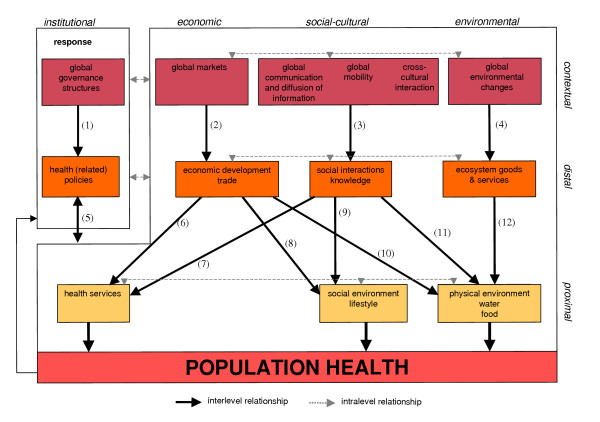
Conceptual model for globalisation and population health.

Figure 3, subsequently, shows that within the developed framework, several links between the specific features of globalisation and health can be derived. These important links between globalisation and health are discussed in the following sections. It is important to note that Figure 3 primarily focuses on the relationships in the direction from globalisation to health. This does not mean, however, that globalisation is an autonomous process: globalisation is influenced by many developments at the other levels, although these associations are not included in the Figure for reasons of simplification. In addition, the only feedback that is included in Figure 3 concerns the institutional response. One also has to keep in mind that determinants within the distal level and within the proximal level also interact with each other, adding complexity to our model (see Huynen et al. 4 for more details and examples of important intralevel relationships).

## Globalisation and distal health determinants

Figure 3 shows that the processes of globalisation can have an impact on all identified distal determinants (Figure 3; arrows 1–4). Below, the implications of the globalisation process on these distal determinants will be discussed in more detail.

### Health(-related) policies

Global governance structures are gaining more and more importance in formulating health(-related) policies (Figure 3; arrow 1). According to Dodgson et al. [8], the most important organisations in global health governance are the World Health Organization (WHO) and the World Bank (WB). The latter plays an important role in the field of global health governance as it acknowledges the importance of good health for economic development and focuses on reaching the Millennium Development Goals [9]. The WB also influenced health(-related) policies together with the International Monetary Funds (IMF) through the Structural Adjustment Programmes (SAPs) (e.g. see Hong [10]). In order to give a more central role to pro-poor growth considerations in providing assistance to low-income countries, the IMF and WB introduced the Poverty Reduction Strategy approach in 1999 [11]. In addition, the policies of the World Trade Organization (WTO) are also increasingly influencing population health [10,12-14]. Fidler [15] argues that 'from the international legal perspective, the centre of power for global health governance has shifted from WHO to the WTO'. Opinions differ with regard to whether the WTO agreements provide sufficient possibilities to protect the population from the adverse (health) effects of free trade or not [16]. In 2002, the WTO ruled that the French ban on the import of all products containing asbestos was legal on health grounds, despite protests from Canada [17,18]. However, protecting citizens against health risks remains difficult, as health standards often need to be supported by sound scientific evidence before trade can be restricted (see e.g. the WTO ruling against the European trade barrier concerning hormone-treated meat [19,20]).

Another important development is the growing number of public-private partnerships for health, as governments increasingly attract private sector companies to undertake tasks that were formerly the responsibility of the public sector. At the global level, public-private partnerships are more and more perceived as a possible new form of global governance [12] and could have important implications for health polices, but also for health-related policies.

### Economic development

Opinions differ with regard to the economic benefits of economic globalisation (Figure 3; arrow 2). On the one side, 'optimists' argue that global markets facilitate economic growth and economic security, which would benefit health. They base themselves on the results of several studies that argue that inequities between and within countries have decreased due to globalisation (e.g. see Frankel [21], Ben David [22], Dollar and Kraay [23]). Additionally, it is argued that although other nations or households might become richer, absolute poverty is reduced and that this is beneficial for the health of the poor [24]. On the other side, 'pessimists' are worried about the health effects of the exclusion of nations and persons from the global market. They argue that the risk of exclusion from the growth dynamics of economic globalisation is significant in the developing world [25]. In fact, notwithstanding some spectacular growth rates in the 1980's, especially in east Asia, incomes per capita declined in almost 70 countries during the same period [26]. Many worry about what will happen to the countries that cannot participate in the global market as successful as others.

### Trade

Due to the establishment of global markets and a global trading system, there has been a continuing increase in world trade (Figure 3; arrow 2). According to the WTO, total trade multiplied by a factor 14 between 1950 and 1997 [27]. Today all countries trade internationally and they trade significant proportions of their national income; around 20 percent of world output is being traded. The array of products being traded is wide-ranging; from primary commodities to manufactured goods. Besides goods, services are increasingly being traded as well [28]. In addition to legal trade transactions, illegal drug trade is also globalising, as it circumvents national and international authority and takes advantage of the global finance systems, new information technologies and transportation.

### Social interactions: migration

Due to the changes in the infrastructures of transportation and communication, human migration has increased at unprecedented rates (Figure 3; arrow 3) [28]. According to Held et al. [28] tourism is one of the most obvious forms of cultural globalisation and it illustrates the increasing time-space compression of current societies. However, travel for business and pleasure constitutes only a fraction of total human movement. Other examples of people migrating are missionaries, merchant marines, students, pilgrims, militaries, migrant workers and Peace Corps workers [28,29]. Besides these forms of voluntary migration, resettlement by refugees is also an important issue. However, since the late 1970s, the concerns regarding the economic, political, social and environmental consequences of migration has been growing and many governments are moving towards more restrictive immigration policies [30].

### Social interactions: conflicts

The tragic terrorist attacks in New York and Washington D.C. in September 2001 fuelled the already ongoing discussions on the link between globalisation and conflicts. Globalisation can decrease the risk on tensions and conflicts, as societies become more and more dependent on each other due the worldwide increase in global communication, global mobility and cross-cultural interactions (Figure 3; arrow 3). Others argue that the resistance to globalisation has resulted in religious fundamentalism and to worldwide tensions and intolerance [31]. In addition, the intralevel relationships at the distal level play a very important role, because many developments in other distal factors that have been associated with the globalisation process are also believed to increase the risk on conflicts. In other words, the globalisation-induced risk on conflict is often mediated by changes in other factors at the distal level [4].

### Social interactions: social equity and social networks

Cultural globalisation (global communication, global mobility, cross-cultural interaction) can also influence cultural norms and values about social solidarity and social equity (Figure 3; arrow 3). It is feared that the self-interested individualism of the marketplace spills over into cultural norms and values resulting in increasing social exclusion and social inequity. Exclusion involves disintegration from common cultural processes, lack of participation in social activities, alienation from decision-making and civic participation and barriers to employment and material sources [32]. Alternatively, a socially integrated individual has many social connections, in the form of both intimate social contacts as well as more distal connections [33]. On the other hand, however, the geographical scale of social networks is increasing due to global communications and global media. The women's movement, the peace movement, organized religion and the environmental movement are good examples of such transnational social networks. Besides these more formal networks, informal social networks are also gaining importance, as like-minded people are now able to interact at distance through, for example, the Internet. In addition, the global diffusion of radio and television plays an important role in establishing such global networks [28]. The digital divide between poor and rich, however, can result in social exclusion from the global civil society.

### Knowledge

The knowledge capital within a population is increasingly affected by developments in global communication and global mobility (Figure 3: arrow 3). The term 'globalisation of education' suggests getting education into every nook and cranny of the globe. Millions of people now acquire part of their knowledge from transworld textbooks, due to the supraterritoriality in publishing. Because of new technologies, most colleges and universities are able to work together with academics from different countries, students have ample opportunities to study abroad and 'virtual campuses' have been developed. The diffusion of new technologies has enabled researchers to gather and process data in no time resulting in increased amounts of empirical data [34]. New technologies have even broadened the character of literacy. Scholte [34] argues that 'in many line of work the ability to use computer applications has become as important as the ability to read and write with pen and paper. In addition, television, film and computer graphics have greatly enlarged the visual dimensions of communication. Many people today 'read' the globalised world without a book'. Overall, it is expected that the above-discussed developments will also improve health training and health education (e.g. see Feachem [24] and Lee [35]).

### Ecosystem goods and services

Global environmental changes can have profound effects on the provision of ecosystem goods and services to mankind (Figure 3; arrow 4). The Intergovernmental Panel on Climate Change (IPCC) [36] concludes that it is expected that climate change can result in significant ecosystem disruptions and threatens substantial damage to the earth's natural systems. In addition, several authors have addressed the link between biodiversity and ecosystem functioning and it is agued that maintaining a certain level of biodiversity is necessary for the proper provision of ecosystem goods and services [37-40]. However, it is still unclear which ecosystem functions are primarily important to sustain our physical health. Basically, the following types of 'health functions' can be distinguished. First, ecosystems provide us with basic human needs like food, clean air, clean water and clean soils. Second, they prevent the spread of diseases through biological control. Finally, ecosystems provide us with medical and genetic resources, which are necessary to prevent or cure diseases [41].

## Globalisation and proximal health determinants

Figure 3 shows that the impact of globalisation on each proximal health determinant is mediated by changes in several distal factors (Figure 3; arrows 5–12). The most important relationships will be discussed in more detail below. It is important to note that health policies and health-related policies can have an influence on all proximal factors (Figure 3; arrow 5).

### Health services

Health services are increasingly influenced by globalisation-induced changes in health care policy (Figure 3; arrow 5), economic development and trade (Figure 3: arrow 6), and knowledge (Figure 3; arrow 7), but also by migration (3: arrow 7). Although the WHO aims to assist governments to strengthen health services, government involvement in health care policies has been decreasing and, subsequently, medical institutions are more and more confronted with the neoliberal economic model. Health is increasingly perceived as a private good leaving the law of the market to determine whose health is profitable for investment and whose health is not [10]. According to Collins [42] populations of transitional economies are no longer protected by a centralized health sector that provides universal access to everyone and some groups are even denied the most basic medical services. The U.S. and several Latin American countries have witnessed a decline in the accessibility of health care following the privatisation of health services [43].

The increasing trade in health services can have profound implications for provision of proper health care. Although it is perceived as to improve the consumer's choice, some developments are believed to have long-term dangers, such as establishing a two-tier health system, movement of health professionals from the public sector to the private sector, inequitable access to health care and the undermining of national health systems [10,12]. The illegal trading of drugs and the provision of access to controlled drugs via the Internet are potential health risks [44]. In addition, the globalisation process can also result in a 'brain-drain' in the health sector as a result of labour migration from developing to developed regions.

However, increased economic growth is generally believed to enhance improvements in health care. Increased (technological) knowledge resulting from the diffusion of information can further improve the treatment and prevention of all kinds of illnesses and diseases.

### Social environment

The central mechanism that links personal affiliations to health is 'social support,' the transfer from one person to another of instrumental, emotional and informational assistance [45]. Social networks and social integration are closely related to social support [46] and, as a result, globalisation-induced changes in social cohesion, integration and interaction can influence the degree of social support in a population (Figure 3; arrow 9). This link is, for example, demonstrated by Reeves [47], who discussed that social interactions through the Internet influenced the coping ability of HIV-positive individuals through promoting empowerment, augmenting social support and facilitating helping others. Alternatively, social exclusion is negatively associated with social support.

Another important factor in the social environment is violence, which often is the result of the complex interplay of many factors (Figure 3; arrows 5, 8 and 9). The WHO [48] argues that globalisation gives rise to obstacles as well as benefits for violence prevention. It induces changes in protective factors like social cohesion and solidarity, knowledge and education levels, and global violence prevention activities such as the implementation of international law and treaties designed to reduce violence (e.g. social protection). On the other hand, it also influences important risk factors associated with violence such as social exclusion, income inequality, collective conflict, and trade in alcohol, drugs or firearms.

### Lifestyle

Due to the widespread flow of people, information and ideas, lifestyles also spread throughout the world. It is already widely acknowledged and demonstrated that several modern behavioural factors such as an unhealthy diet, physical inactivity, smoking, alcohol misuse and the use of illicit drugs are having a profound impact on human health [49-52] (Table 3). Individuals respond to the range of healthy as well as unhealthy lifestyle options and choices available in a community [53], which are in turn determined by global trade (Figure 3; arrow 8), economic development (Figure 3; arrow 8) and social interactions (Figure 3; arrow 9).

**Table 3:** Lifestyle and health

**Lifestyle factor**	**Health effects**
Diet	Excess energy intake results, together with physical activity, in obesity. Obesity is an increasing health problem and has several co-morbidities such as non-insulin dependent diabetes and cardiovascular diseases [49]. The nutritional quality of the diet (e.g. fruit and vegetable intake, saturated versus unsaturated fats) is also very important for good health.
Inactivity	Physical inactivity has been linked to obesity, coronary hearth disease, hypertension, strokes, diabetes, colon cancer, breast cancer and osteoporotic fractures [49].
Smoking	Tobacco is predicted to be the leading health risk factor by 2030 [50]. It causes, for example, cancer of the trachea, bronchus and lung [49], and cardiovascular diseases.
Alcohol use	The consumption of alcoholic beverages increases to risk on liver cirrhosis, raised blood pressure, heart disease, stroke, pancreatitis and cancers of the oropharnix, larynx, oesophagus, stomach, liver and rectum [49]. The role of alcohol consumption in non-communicable disease epidemiology is, however, complex. For example, small amounts of alcohol reduce the risk on cardiovascular diseases, while drinking larger amounts is an important cause of these very same diseases [51].
Illicit drugs	According to the World Health Report 2001 [52], 0,4 % of the total disease burden is attributable to illicit drugs (heroin and cocaine). Opiate users can have overall mortality rate up to 20 percent higher than those in the general population of the same age, due to not only overdoses but also to accidents, suicides, AIDS and other infectious diseases [49].

Although the major chronic diseases are not transmittable via an infectious agent, the behaviours that predispose to these diseases can be communicated by advertising, product marketing and social interactions [54]. Global trade and marketing developments drive, for example, the nutrition transition towards diets with high proportions of salt, saturated fat and sugars [51,53]. Another example is the worldwide spread of tobacco consumption as transnational tobacco companies take advantage of the potential for growth in developing countries [51,55]. Additionally, the scale of cigarette smuggling poses a considerable global threat to the efforts to control tobacco consumption [44]. Illegal trade in illicit drugs poses similar problems. At the same time, the alcohol industry is almost as globalised as the tobacco industry [56].

However, health education can play a role in promoting healthy lifestyles by improving an individual's knowledge about the health effects of different lifestyle options (Figure 3; arrow 9). Besides health education, (global) policies can also directly discourage unhealthy behaviour by means of economic incentives (e.g. charging excise on tobacco) or other legislation (Figure 3; arrow 5).

### Physical environment: infectious diseases pathogens

The spread of infectious diseases is probably one of the most mentioned health effects of globalisation and past disease outbreaks have been linked to factors that are related to the globalisation process (see e.g. Newcomb [57]). The recent outbreak of the Severe Acute Respiratory Syndrome (SARS) demonstrates the potential of new infectious diseases to spread rapidly in today's world, increasing the risk of a global pandemic. The combination of movement of goods (Figure 3; arrow 10) and people (Figure 3; arrow 11), and profound changes affecting ecosystem goods and services (Figure 3; arrow 12) all contribute to increased risk of disease spread [57]. For example, the globalisation of food production, trade and consumption has been associated with the increased spread and transmission of food born diseases [57,58]. Diseases like HIV/AIDS or hepatitis B can also spread through trade in infected biological products (e.g. blood) [44].

Enhanced knowledge and new technologies will improve the surveillance of infectious diseases and monitoring of antibiotic resistance [24,35] (Figure 3; arrow 11). Globalisation potentially increases the speed of responses in some cases. Wilson [29] states that responding to disease emergence requires a global perspective-both conceptually and geographically-as the current global situation favours the outbreak and rapid spread of infectious disease. As a result, the policies and actions undertaking by the WHO are becoming increasingly important in controlling infectious diseases at a global level (Figure 3; arrow 5). For instance, the WHO played a critical role in controlling SARS by means of global alerts, geographically specific travel advisories and monitoring [59].

### Food

Food trade has become an increasingly important factor with regard to food security worldwide (Figure 3; arrow 10). At present, however, the developed countries usually subsidise their agricultural sectors. Current liberalisation policies are expected to have profound implications on food trade and, subsequently food security [60]. Some argue that the resulting free trade will create access to better and cheaper food supplies via food imports and can stimulate more efficient use of the world's resources as well as the production of food in regions that are more suitable to do so [60,61]. Free trade permits food consumption to grow faster than domestic food production in countries where there are constraints on increasing the latter. Accelerated economic growth can also contribute to food security (Figure 3; arrow 10) [60]. Others, however, argue that the forces of globalisation in fact endanger food security (e.g. see Lang [62]) and that countries should strive to become more self-sufficient [60]. For many countries the increasing dependence on food imports goes hand in hand with a higher vulnerability to shocks arising in global markets, which can affect import capacity and access to food imports [60]. Many food insecure countries are not able to earn enough with exporting goods in order to pay for the needed food imports [63].

At the global level, there are increasing international efforts to achieve widespread food security (Figure 3; arrow 5). For instance, the right to adequate food is directly addressed in the 1966 International Covenant on Economic, Social and Cultural Rights. In 1996, the World Food Summit reaffirmed the right of everyone to have access to safe and nutritious food. In case of extreme food-insecurity and insufficient import capacity, food aid may be provided in order to supplement the scarce food imports. Globalisation can affect food security by enhancing the knowledge of foreign nations about the usefulness of food aid (Figure 3; arrow 11) [60].

Besides food trade, one can also deal with the mismatch between demand and supply by increasing food production in food-short regions. The globalisation process can increase food security by facilitating the worldwide implementation of better technologies and improved knowledge (e.g. irrigation technologies, research on genetically modified food) (Figure 3; arrow 11). At the same time, the natural resource base for food production is increasingly threatened (Figure 3; arrow 12). Finally conflicts are, of course, a threat to food security and it is expected that food security in sub-Saharan Africa, for example, will not increase without the establishment of political instability (Figure 3; arrow 11) [64].

### Water

The effects of globalisation are also raising concerns over water security. The current globalisation process is accompanied by privatisation policies affecting the provision of water [65] (Figure 3; arrow 5). Governments and international financial institutions promote privatisation, as they believe it will promote market competition and efficiency. However, others are less optimistic about the effects of privatisation. In fact, some cases show that prices and inequalities in access even rise [66]. It is also argued that water, with vital importance socially, culturally, and ecologically, 'cannot be protected by purely market forces' [65]. On a global scale, there are increasing efforts to set up global guidelines or policies with regard to fresh water (Figure 3; arrow 5), however none of the international declarations and conference statements requires states to actual meet individual's water requirements [67].

The virtual trade of water is also believed to be of increasing importance (Figure 3; arrow 10). The water that is used in the production process of a commodity is called the 'virtual water' contained in that commodity. Therefore, the increasing global trade of commodities is accompanied by an increasing global trade in virtual water. The global volume of virtual water embedded in crop and livestock products traded between nations is estimated to be 1400 billion cubic metres per year [68].

In addition, the globalisation process can increase water security by facilitating the worldwide implementation of better technologies and improved knowledge (Figure 3; arrow 11). At the same time, the natural resource base is increasingly threatened as, for example, global climate change and deforestation profoundly affect our ecosystems ability to provide us with sufficient and adequate fresh water (Figure 3; arrow 12).

## Conclusion

Globalisation is causing profound and complex changes in the very nature of our society, bringing new opportunities as well as risks. In addition, the effects of globalisation are causing a growing concern for our health, and the intergenerational equity implied by 'sustainable development' forces us to think about the right of future generations to a healthy environment and a healthy life.

Despite some empirical research efforts indicating the links between the globalisation process and specific health impacts, the present weakness in empirical evidence on the multiple links between globalisation and health is still a problem [44]. The described conceptual framework could give a meaningful contribution to further empirical research by serving as a well-structured 'think-model' or 'concept map'. It clearly demonstrates that an interdisciplinary approach towards globalisation and health is required, which draws upon the knowledge from relevant fields such as, for example, medicine, epidemiology, sociology, political sciences, (health) education, environmental sciences and economics.

In addition, the exploration of possible future health impacts of different globalisation pathways by means of scenarios analysis could provide a useful contribution to the ongoing discussions on globalisation and health [4]. Scenarios can be described as 'plausible but simplified descriptions of how the future may develop, according to a coherent and internally consistent set of assumptions about key driving forces and relationships' [69]. Recent research showed, however, that the health dimension is largely missing in existing global scenarios [70]. The developed framework for globalisation and population health has contributed to the understanding of future health implications and the model is, therefore, considered to be a useful tool to structure future scenario studies on the health implications of the globalisation process.

To conclude, the framework provides valuable insights in how to organise the complexity involved in studying the health effects resulting from globalisation. We claim that our approach has several beneficial characteristics. First, it is embedded in a holistic approach towards globalisation; in this paper we perceive globalisation as an overarching process in which simultaneously many different processes take place in many societal domains. In addition, the conceptual framework is embedded in a holistic approach towards population health. As a result, our model explicitly visualises that globalisation affects the institutional, economic, social-cultural and ecological determinants of population health and that the globalisation process mainly operates at the contextual level, while influencing health through the more distal and proximal determinants.

## Competing interests

The author(s) declare that they have no competing interests.
